# Statistical shape modeling of the talocrural joint using a hybrid multi-articulation joint approach

**DOI:** 10.1038/s41598-021-86567-7

**Published:** 2021-04-01

**Authors:** Amy L. Lenz, Nicola Krähenbühl, Andrew C. Peterson, Rich J. Lisonbee, Beat Hintermann, Charles L. Saltzman, Alexej Barg, Andrew E. Anderson

**Affiliations:** 1 Department of Orthopaedics, University of Utah, Salt Lake City, 84108 USA; 2 Department of Orthopaedics, Kantonsspital Baselland, 4410 Liestal, Switzerland; 3 Department of Orthopaedics, Trauma and Reconstructive Surgery, University Medical Center Hamburg-Eppendorf, 20251 Hamburg, Germany

**Keywords:** Anatomy, Medical research, Mathematics and computing

## Abstract

Historically, conventional radiographs have been the primary tool to morphometrically evaluate the talocrural joint, which is comprised of the distal tibia, distal fibula, and proximal talus. More recently, high-resolution volumetric imaging, including computed tomography (CT), has enabled the generation of three-dimensional (3D) reconstructions of the talocrural joint. Weightbearing cone-beam CT (WBCT) technology provides additional benefit to assess 3D spatial relationships and joint congruency while the patient is load bearing. In this study we applied statistical shape modeling, a computational morphometrics technique, to objectively quantify anatomical variation, joint level coverage, joint space distance, and congruency at the talocrural joint. Shape models were developed from segmented WBCT images and included the distal tibia, distal fibula, and full talus. Key anatomical variation across subjects included the fibular notch on the tibia, talar trochlea sagittal plane rate of curvature, tibial plafond curvature with medial malleolus prominence, and changes in the fibular shaft diameter. The shape analysis also revealed a highly congruent talocrural joint with minimal inter-individual morphometric differences at the articular regions. These data are helpful to improve understanding of ankle joint pathologies and to guide refinement of operative treatments.

## Introduction

The talocrural joint is comprised of the articular relationships between the tibia, fibula and talus, including three main articular regions (tibiotalar, tibiofibular and talofibular)^1^. Morphometric understanding of the ankle joint complex has been derived primarily from two-dimensional (2D) measurements of conventional radiographs, with little focus given to the fibula and the complete talocrural joint^2–5^. Numerous 2D measurements have been developed in attempt to quantify normal and pathological anatomy within the ankle joint, distal syndesmosis and medial/lateral gutter^4–6^. However, these complex three-dimensional (3D) morphologies are not adequately represented in single 2D measurements that are most often isolated to a single imaging plane^7^.

Volumetric imaging, such as computed tomography (CT), has made it possible to generate 3D reconstructions of the talocrural joint^8,9^. Weightbearing cone-beam computed tomography (WBCT) is an emerging volumetric imaging technology that provides an added benefit to assess pathology, joint space narrowing, and loss of joint congruency that may be overlooked in the absence of load^10,11^. Early studies using WBCT established joint space distance relationships of the ankle joint, but analyses were primarily focused on the tibiotalar articular regions^12,13^. Furthermore, joint space analysis was not paired with evaluation of bone morphology variation, articular coverage within the joint, or the congruency of mated articular surfaces. An improved understanding of the ankle joint morphometrics is crucial for continued development of surgical treatment strategies. As an example, surgeries for early-stage ankle osteoarthritis, such as osteotomies, primarily aim to correct underlying anatomical deformities to improve ankle loading and kinematic motion and provide pain relief.

Statistical shape modeling (SSM) can be used to visualize and analyze morphological differences in 3D^14^. SSM enables the identification of mean bone shapes and shape modes of variation using correspondence particles (mathematically placed points of interest throughout the bone’s surface) which eliminates human bias^15,16^. Previous studies applying SSM in the foot and ankle have assessed bone variation independent of joint relationships^17–20^. The development of a joint level SSM analysis is needed to assess bone variation relative to joint articular relationships. The objective of the present study was to develop a talocrural joint level SSM to evaluate shape variances in combination with joint coverage, distance and congruency based on cone-beam WBCT scans to describe healthy talocrural 3D morphology. The presented model should serve as a reference for future research focusing on ankle or hindfoot pathologies.

## Results

### Talocrural joint shape variation

#### Tibial shape variation

**Figure 1:**
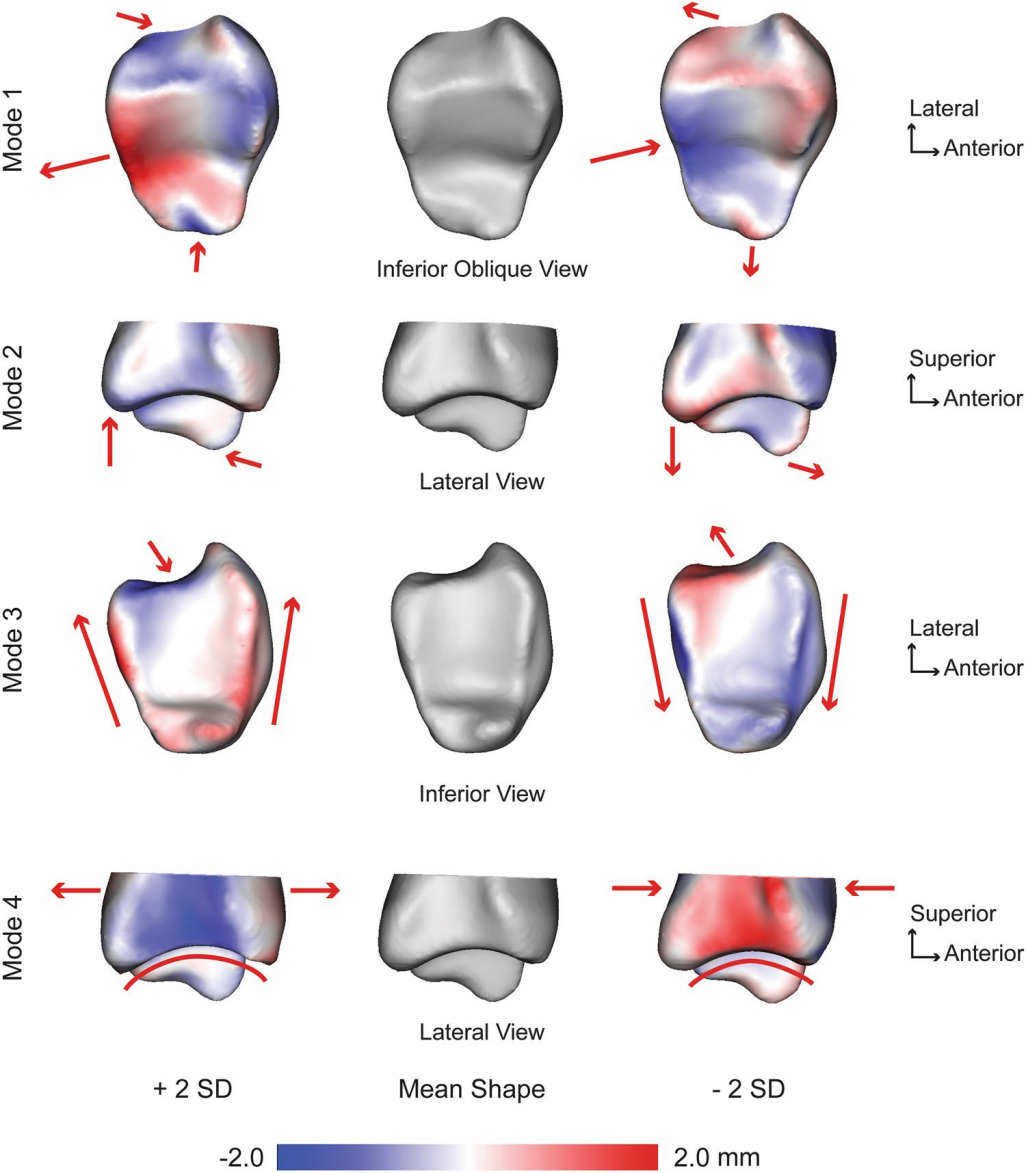
Tibial modes of variation 1–4 showing significant anatomical differences observed at ± 2 standard deviations (SD) from the mean shape (center; grey). Red arrows focus on the location and direction of change for key anatomical features (pointing at the bone indicates the decreased size of a feature, whereas an arrow pointing away from the bone indicates an increased size of a feature). Surface distances shown on ± 2 SD represent the distance away from the mean shape with a scale in millimeters (mm).

For the tibia, seven PCA modes were significant that described 78.2% of the overall shape variation. The individual seven modes (1–7) contained 31.9%, 13.5%, 9.8%, 9.2%, 5.6%, 4.2%, and 4.0% of the significant variation, respectively. Anatomical variations were observed across each of these significant tibial modes. The first mode of variation consisted of numerous anatomical differences across ± 2 standard deviations (SD). Notably, at + 2 SD, there was increased inferior height of the medial malleolus, equal posterior and anterior prominences of tibial plafond, decreased medial/lateral depth of the fibular notch, and increased overall medial/lateral width in the axial plane. At -2 SD, there was a decreased inferior height of the medial malleolus, a more prominent posterior prominence of the tibial plafond, increased medial/lateral depth of the fibular notch, and decreased overall medial/lateral width in the axial plane (Fig. 1). The second mode of variation included similar features with the addition of variance of the anterior/posterior length of the tibial plafond, corresponding with a varying rate of curvature (Fig. 1). Remarkable differences in the third mode of variation included an increased lateral aspect anterior/posterior distance with a deeper fibular notch versus a decreased lateral aspect anterior/posterior distance with a shallower fibular notch. With little differences seen on the medial aspect, this led to a trapezoidal versus rectangular shape for the tibial plafond (Fig. 1). The fourth mode of variation consisted of an increased anterior distance which corresponded with a sharper radius of curvature of the tibial plafond versus a decreased anterior distance with a broader radius of curvature (Fig. 1). The fifth mode of variation represented a varying fibular notch, anterior/posterior distance and medial malleolus. The sixth mode of variation was similar to the first mode of variation without demonstrating variation in the anterior and posterior prominences of the tibial plafond. Lastly, the seventh mode of variation included medial malleolus height changes and increased posterior prominence of the tibial plafond.

#### Fibular shape variation

**Figure 2:**
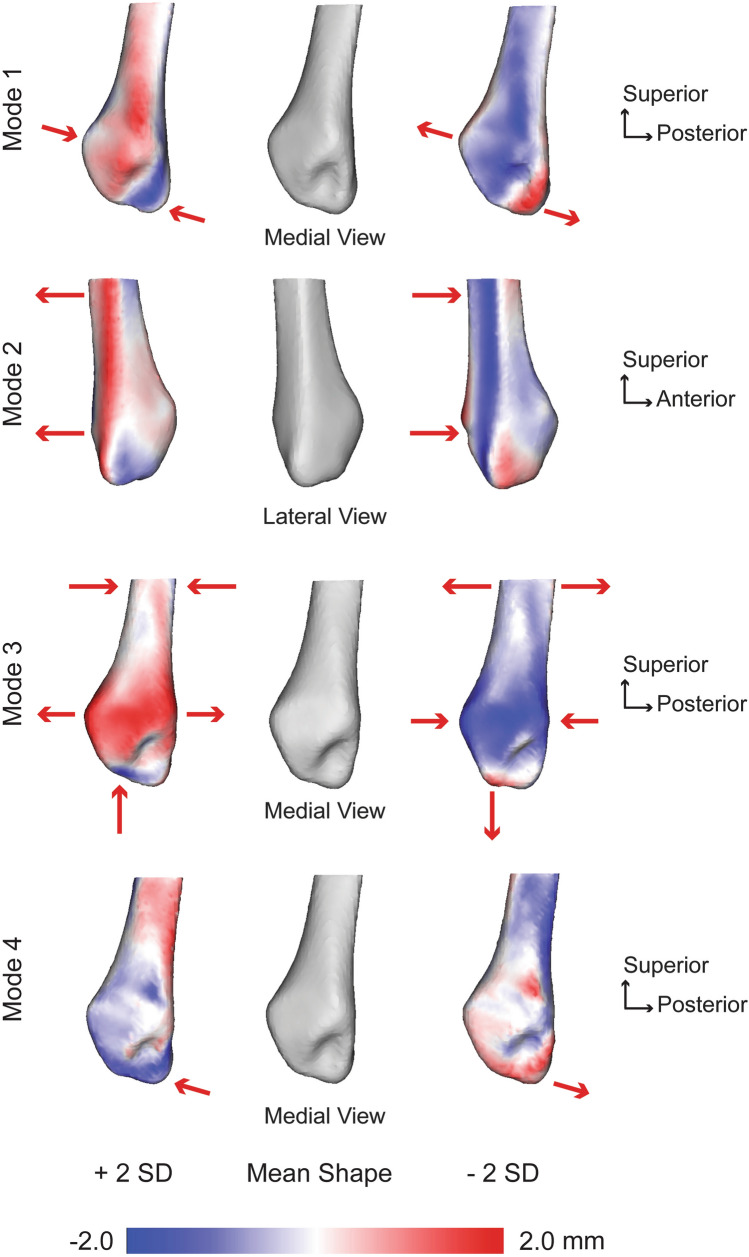
Fibular modes of variation 1–4 showing significant anatomical differences observed at ± 2 standard deviations (SD) from the mean shape (center; grey). Red arrows focus on the location and direction of change for key anatomical features (pointing at the bone indicates the decreased size of a feature, whereas an arrow pointing away from the bone indicates an increased size of a feature). Surface distances shown on ± 2 SD represent the distance away from the mean shape with a scale in millimeters (mm).

For the fibula, seven PCA modes were significant that described 74.8% of the overall shape variation. The individual seven modes (1–7) contained 20.6%, 16.8%, 12.2%, 9.2%, 6.1%, 5.3%, and 4.6% of the significant variation, respectively. Anatomical variations were observed across each of these significant fibular modes. The first mode of variation consisted of variance in the malleolar fossa and angulation of the articular facet of the lateral malleolus (Fig. 2). A deeper malleolar fossa corresponded with an increased anterior/posterior width of the articular facet, decreased medial/lateral width from the articular facet to the lateral aspect of the malleolus, and a reduced articular facet angulation towards the distal malleolus at -2 SD for this first mode. Opposite of this at + 2 SD, a shallower malleolar fossa corresponded with a decreased anterior/posterior width of the articular facet, increased medial/lateral width, and an increased articular facet angulation. The second mode of variation primarily consisted of a posterior versus anterior shift of the lateral spine on the fibula (Fig. 2). A posterior-shifted spine (for peroneal tendon containment) corresponded with a decreased anterior/posterior width of the articular facet, whereas an anterior spine correlated with an increased articular facet width. The third mode of variation showed an increased distal malleolar prominence with a flatter and narrower articular facet and a wider proximal shaft versus a reduced distal malleolar prominence with a curved and broader articular facet and narrower proximal shaft (Fig. 2). The fourth mode of variation demonstrated variability surrounding the malleolar fossa curvature and width on the posterior aspect of the fibula (Fig. 2). The fifth mode of variation showed a widening articular facet and depth changes in the malleolar fossa. The sixth mode of variation showed primary features consisting of distal prominence variation, medial/lateral width and articular facet articulation. Lastly, the seventh mode of variation included changes in articular facet width, fossa depth, and angulation of the facet.

#### Talar shape variation

**Figure 3:**
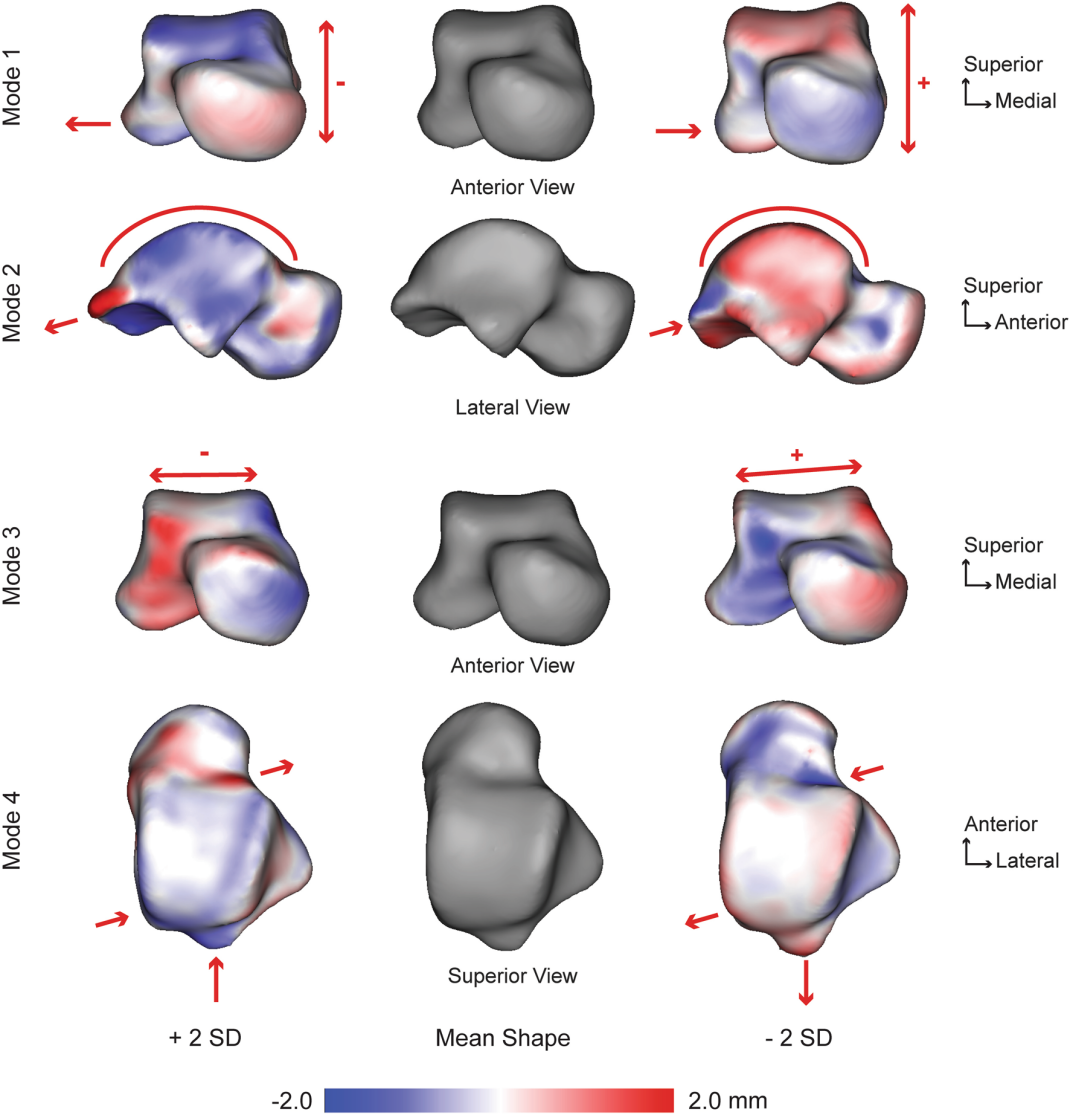
Talar modes of variation 1–4 showing significant anatomical differences observed at ± 2 standard deviations (SD) from the mean shape (center; grey). Red arrows focus on the location and direction of change for key anatomical features (pointing at the bone indicates the decreased size of a feature, whereas an arrow pointing away from the bone indicates an increased size of a feature). Surface distances shown on ± 2 SD represent the distance away from the mean shape with a scale in millimeters (mm).

For the talus, seven PCA modes were significant that described 65.5% of the overall shape variation. The individual seven modes (1–7) contained 16.5%, 13.4%, 11.0%, 8.7%, 6.0%, 5.4%, and 4.5% of the significant variation, respectively. Anatomical variations were observed across each of these significant tibial modes. The first mode of variation consisted of an overall increase and decrease of the superior/inferior talar height which corresponded with a more anterior prominent shift of the talar trochlea and decreased lateral process with increased talar body height (Fig. 3). The second mode of variation consisted of a varying anterior/posterior distance from the talar neck to the posterior process. A shorter anterior/posterior distance corresponded with talar trochlea smaller rate of curvature, deeper talar neck, and a decreased posterior process prominence (Fig. 3). The third mode of variation was primarily described by a varying medial/lateral distance of the talar trochlea, where a wider talar body through the talar trochlea corresponded with a deeper and higher rate of frontal plane curvature of the articular surface and a downward slope to the lateral aspect (Fig. 3). The fourth mode of variation consisted of a posterior/medial shift of the talar trochlea surface area, which corresponded with a deeper talar neck and an increased posterior process prominence (Fig. 3). The fifth mode of variation primarily demonstrated frontal plane differences in the talar trochlea width. The sixth mode of variation primarily consisted of overall talar dome height differences and depth changes of the talar neck. Lastly, the seventh mode of variation was a subtle combination of multiple modes of variation with the primary feature being the talar dome width differences with slight changes in height.

### Talocrural joint coverage, distance and congruency in a weightbearing neutral position

#### Tibiotalar articulation

**Figure 4:**
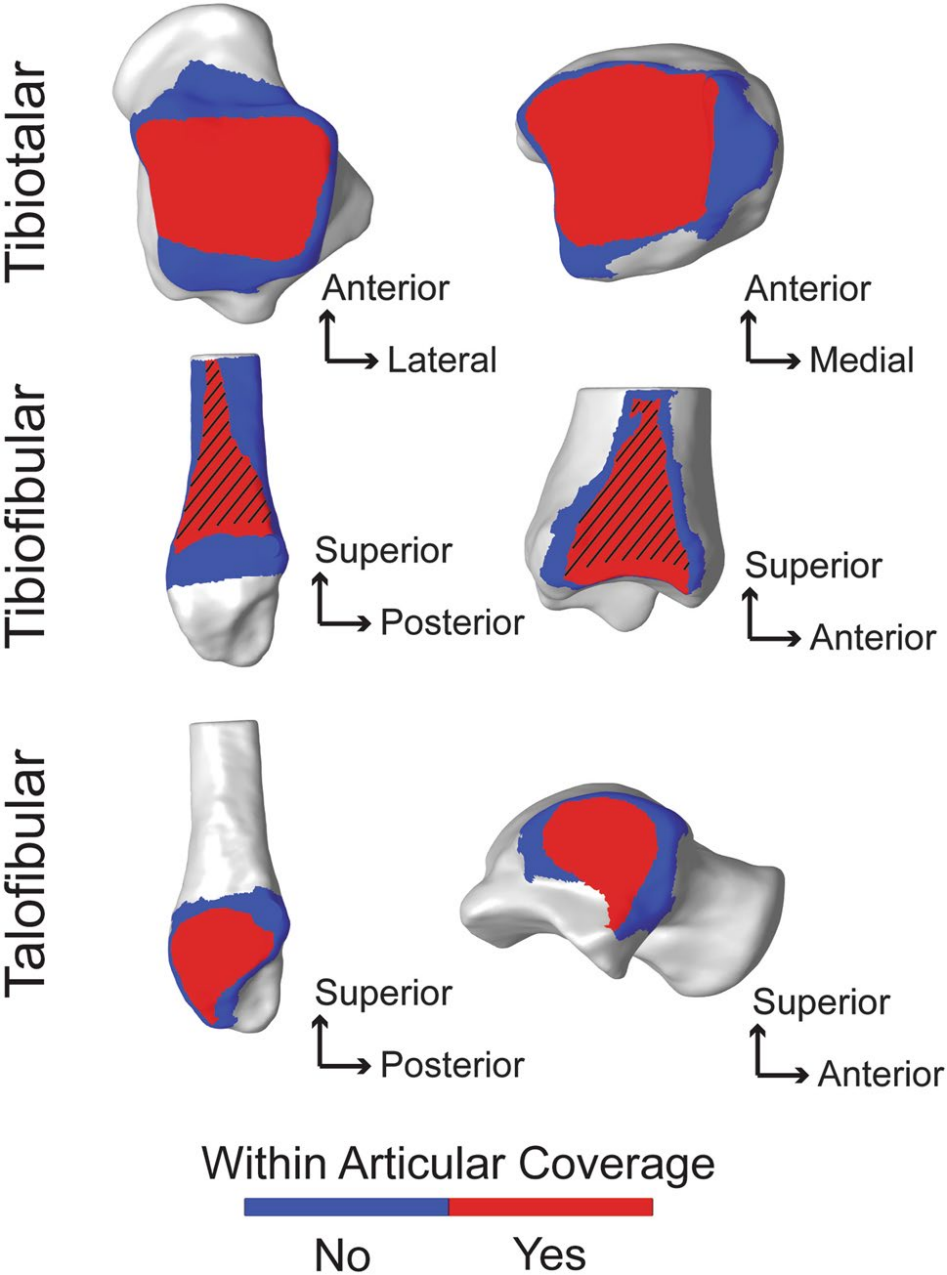
Coverage analysis showing regions of articular coverage for the tibiotalar, tibiofibular and talofibular joints. Regions within the articular coverage are shown in red. Regions in blue represent the initial region of selection based on the second principal curvature, with the rest of the bone shown in grey. Important to note, the articular coverage regions are defined mathematically as described in the methods and due to the imaging limitation of WBCT cannot be directly correlated with regions containing articular cartilage. However, the region for the tibiofibular relationship, shown with the black angled lines, represents the region of the distal tibiofibular syndesmosis anatomically known to contain the interosseous ligament throughout this generalized region. Distal to the dashed region, at the base of the syndesmosis, there is typically a small tibiofibular contact zone^32^. But for the purpose of this analysis, the tibiofibular contact zone and syndesmosis regions were not separated mathematically within the articular coverage region identified.

**Figure 5:**
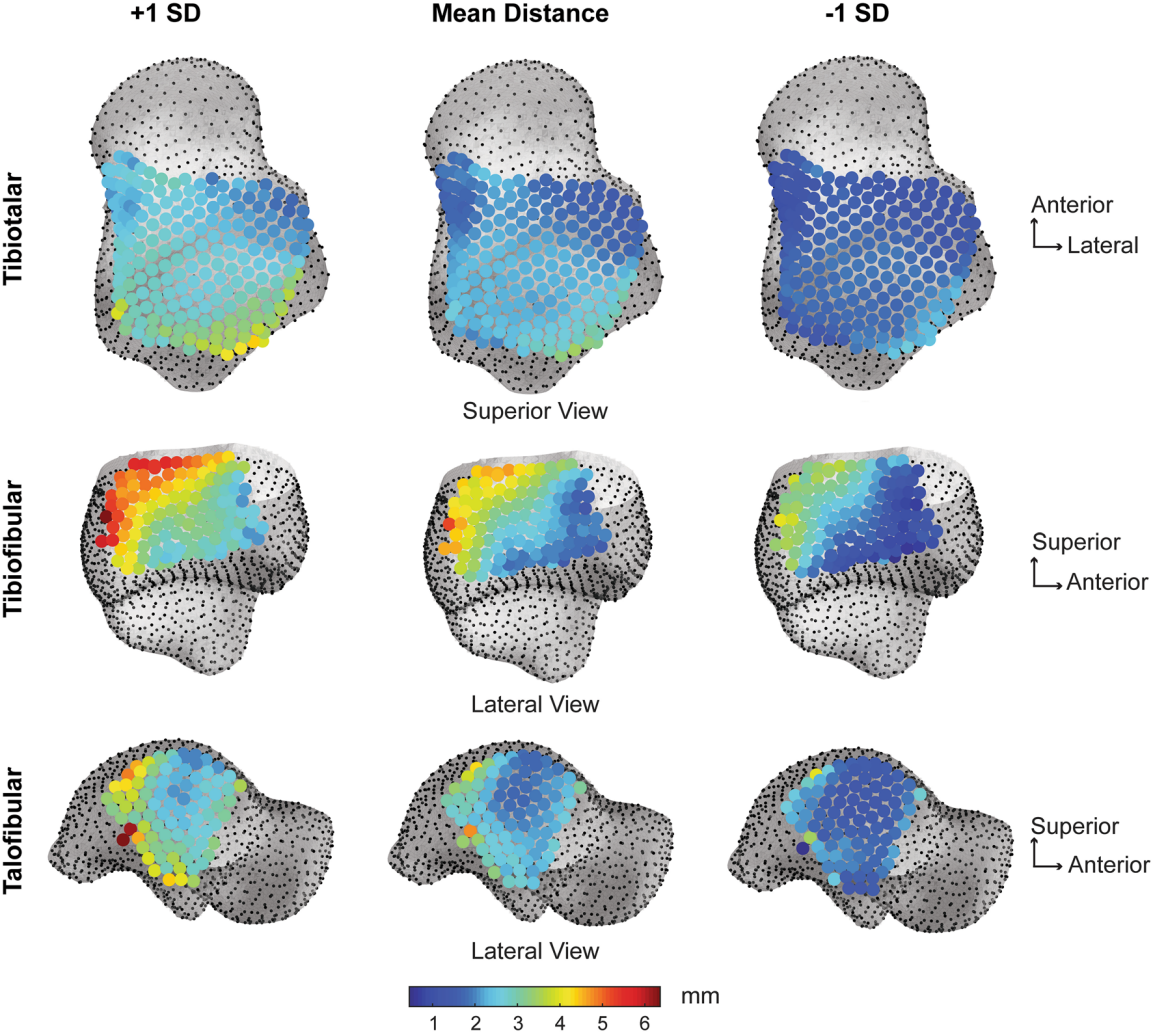
Average joint distance space with ± 1 standard deviation (SD) for the tibiotalar, tibiofibular and talofibular articular regions. Results are visualized on the mean shape correspondence particles.

**Figure 6:**
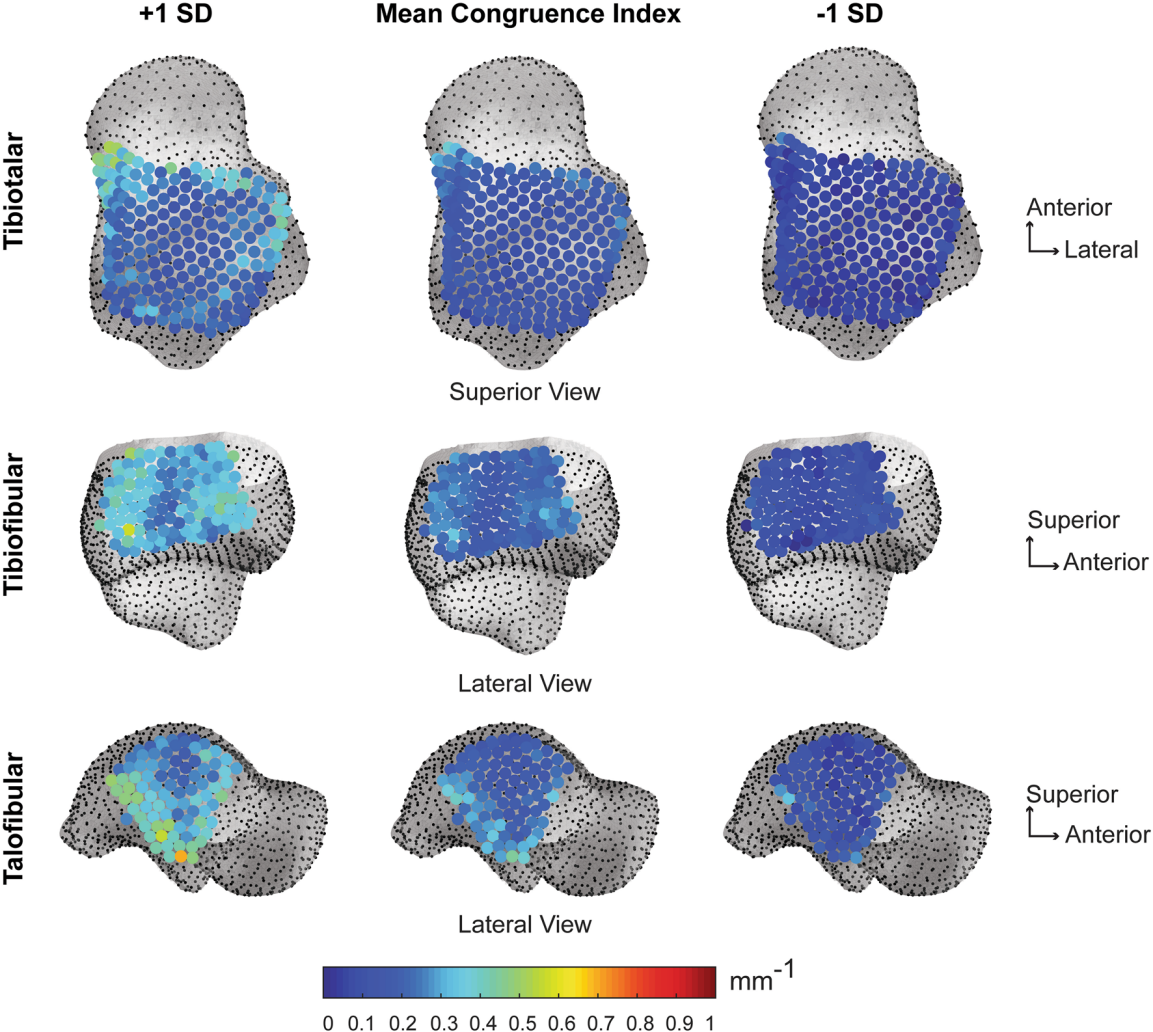
Average joint congruence index with ± 1 standard deviation (SD) for the tibiotalar, tibiofibular and talofibular articular regions. Results are visualized on the mean shape correspondence particles. Congruence index rated with a starting value of 0 mm^-1^, with 0 demonstrating perfect congruency.

Tibiotalar joint articular coverage surface area values were significantly different between the tibial and talar regions (Fig. 4). The tibial plafond surface area was significantly greater than the talar trochlea surface area by an average of 151.32 mm^2^, p = 0.0001, Cohen’s d = 1.61, post hoc power = 97.5% (Table 1). The tibiotalar joint space distance was relatively even with an average distance of 2.15 mm (Table 2). The narrowest joint space distance was ~ 1 mm in the anterolateral region, whereas the widest joint space was ~ 3.5 mm in the posterolateral region (Fig. 5). Congruency of the tibiotalar articulation was even throughout with an average congruence index of 0.15 ± 0.05 mm^-1^ (Table 2). Across the healthy population studied, the tibiotalar congruence index was highly consistent with slight variation shown in the anteromedial region (Fig. 6).

**Table 1:** Average surface area ± standard deviation (SD) reported for the coupled articular surfaces within the coverage region for the tibiotalar, tibiofibular and talofibular articulations.

Joint	Coverage (Average ± SD)			
	Tibia (mm^2^)	Talus (mm^2^)	Fibula (mm^2^)	p-value
Tibiotalar	943.21 ± 103.64	791.89 ± 83.35	–	*p* = *0.001*
Tibiofibular	570.70 ± 83.55	–	454.00 ± 65.47	*p* = *0.001*
Talofibular	–	320.28 ± 61.74	316.91 ± 67.97	p = 0.376

**Table 2:** Joint distance space and congruence index reported as an average ± standard deviation (SD), with minimum (Min) and maximum (Max) values for the tibiotalar, tibiofibular, and talofibular articular regions.

Joint	Joint distance (mm)			Congruence index (mm^−1^)		
	Ave ± SD	Min	Max	Ave ± SD	Min	Max
Tibiotalar	2.15 ± 0.41	1.08	3.46	0.15 ± 0.05	0.06	0.38
Tibiofibular	3.36 ± 0.83	2.00	5.38	0.21 ± 0.04	0.12	0.35
Talofibular	2.43 ± 0.56	1.69	4.77	0.22 ± 0.07	0.09	0.44

#### Tibiofibular articulation

Tibiofibular joint articular coverage surface area values were significantly different between the tibial and fibular regions (Fig. 4). The tibial surface area within the fibular notch was significantly greater than the mated fibular surface area by an average of 116.70 mm^2^, p = 0.0001, Cohen’s d = 1.55, post hoc power = 96.7% (Table 1). The tibiofibular joint space distance demonstrated an uneven gradient, with widest distance shown posteriorly (~ 5 mm) and gradually narrowing towards the anterior region (~ 2 mm; Fig. 5). On average the tibiofibular joint space distance was 3.36 mm (Table 2). Congruency of the tibiofibular articulation was most consistent in the middle of the fibular notch, with slight variation in the anterior and poster regions (Fig. 6). The average congruence index was 0.21 ± 0.04 mm^-1^ (Table 2).

#### Talofibular articulation

Talofibular joint articular coverage surface area values were not significantly different between the talar and fibular regions, p = 0.376, Cohen’s d = 0.05 (Fig. 4). The talar articular surface area for the lateral malleolus was on average only 3.37 mm^2^ larger than the fibular malleolar articular surface area and a post hoc power analysis revealed that in order for a large effect to be detected (d = 0.8) with an α = 0.05, a sample size of 4840 participants would be required (Table 1). The talofibular joint space distance was more variable with an average distance of 2.43 mm (Table 2). The narrowest and most consistent joint space distance was shown in the middle of the articular surface (Fig. 5). Congruency of the talofibular articulation showed highest variation in the posterior articular border (Fig. 6). The average congruence index was 0.22 ± 0.07 mm^−1^ (Table 2).

## Discussion

SSM has achieved considerable success in quantifying morphology from segmented medical images. Early SSM of the bones of the ankle were performed on 2D radiographs using manual landmark selection^21^. Advancements in 3D medical imaging and development of SSM algorithms have expanded the morphology literature in the foot and ankle^9,17–19,22–24^. Yet, a large limitation of SSM remains to be a lack of clinical interpretation of the PCA modes of variation. Further, limited clinical models to date have combined an individual bone SSM with joint level analyses of the articular surface relationships. Therefore, studies presenting only the mathematical model output without linking results to anatomical features can make it difficult to compare SSM results in a clinically meaningful manner^19,22,23^. Our study addresses this gap in knowledge through a comprehensive shape and multi-articulation analysis for the complete talocrural joint.

Our presented tibial, fibular, and talar shape variations correspond with the limited literature available, which highlighted anatomical variation of the fibular notch on the tibia, talar trochlea sagittal plane rate of curvature, tibial plafond curvature with medial malleolus prominence, and changes in the fibular shaft diameter^17,18,20,24^. While our models had the limitation of not including the full-length tibia and fibula in our SSM analysis, we gained the ability to have higher resolution imaging in a weightbearing position to focus on more shape variation details within the distal tibia and fibula with weightbearing articular relationships and analyses in the talocrural joint. Previous literature has used 3D imaging with surface reconstruction to assess the radius of curvature in the talocrural joint^25–27^. Morphological differences of talar trochlea sagittal plane rate has been identified to impact tibiotalar stability with a flatter talar trochlea being less stable^28^. However, this finding is based on measurements performed on conventional radiographs (lateral view), which may not adequately reflect the complex 3D anatomy of the tibiotalar joint^28^. In addition, the morphology of the incisura fibularis on the tibia has been shown to impact reduction success in syndesmotic injuries^29^. Normative values presented here may help surgeons to better identify patients with a more flat/curved incisura which are at risk for anterior/posterior malreduction of the fibula^29^.

Decreasing joint space distance has been the primary indicator of joint degeneration resulting from osteoarthritis. However, these measures originated by evaluating limited 2D measurements on radiographs, which did not provide a comprehensive distance mapping of the entire articular joint space^30–32^. Recently, Lintz et. al analyzed the distance mapping of the foot and ankle joints in patients with cavovarus deformity^33^. However, there is a need to establish normative joint space distance values based on WBCT to evaluate patient populations. Limited studies have evaluated talocrural joint space using 3D bone models. One cadaver study reported tibiotalar and talofibular articular joint space distances that were subdivided into 9 regions for the talar dome/tibial plafond articulations and two regions within the talofibular articulation^34^. We found joint space distances that were similar, albeit slightly larger, than results reported in this previous cadaver study; this was expected, as the samples in the cadaver study were from donors that were slightly older than participants in our study (50.0 years old in our study versus 53.6 years in ^34^). Our joint space results also agree with an in vivo study which imaged ten healthy ankles in a non-weightbearing position^12^. Tibial plafond joint space distance was reported as 2.04 ± 0.29 mm compared to our tibiotalar joint space distance of 2.15 ± 0.41 mm and talofibular joint space distance was 2.13 ± 0.20 mm compared to our reported 2.43 ± 0.56 mm ^12^. An important point to note when comparing these previous studies to our joint space results include the presence of in vivo weightbearing imaging with the use of a coverage analysis to identify the articular region of interest. In CT imaging, it is not possible to identify an accurate cartilage boundary without contrast injected into the joint space. Thus, we used a mathematical analysis of joint coverage to determine the articular joint space region. It is possible that our average data is slightly higher due to the perimeter selection of our area not being identical to previous studies. However, the use of non-weightbearing analyses and cadaver samples instead of living participants in these prior studies likely explains the cause for differences in reported results.

Quantifying curvature morphology in 3D has proven difficult with complex joint geometries. To our knowledge, our study is the first to report 3D congruency measurements of the talocrural joint. Previously a single cadaver study assessed the curvature of the tibiotalar joint with matched degrees of congruity, but this was performed on excised bones with a grid system of etched marks without the use of high-resolution 3D imaging, making it difficult to directly compare results^35^. Our congruency results can serve as a normative dataset for future comparisons to pathological populations which may have severe incongruity due to pilon fractures, osteoarthritis or hindfoot deformity^36–39^. This possibly will help to better understand the evolution of certain ankle or hindfoot diseases like posttraumatic ankle osteoarthritis or pes cavovarus/planovalgus deformity.

Within the limited literature currently available, we were able to directly compare our talar results to those of Grant et al.^20^ We found slightly more PCA modes that described talus shape. We believe these differences arise for three primary reasons. First, their shape modeling results included size as the primary first mode of variation, whereas we used Procrustes to remove size as the first mode of variation. Second, the shape modeling analysis employed by Grant et al. was based on surface segmentations from magnetic resonance imaging (MRI). Due to the fundamental imaging differences in visualization of cortical bone between MRI versus CT, this may also contribute to slight differences in the surface inputs used to generate the shape models, and therefore the resulting PCA modes. Lastly, Grant et al. used X, Y, Z point clouds of the full bone segmentations and X, Y, Z coordinates of manually selected sparse anatomical landmarks to reconstruct and conduct principal component analyses. Whereas we used ShapeWorks^14^, which establishes hierarchically placed correspondence using a computational optimization splitting strategy^14^. Thus, the differing imaging and computational approaches between our study and Grant et al. likely explain slight differences in the number of PCA modes found to describe talar morphology.

Our study has limitations. First, all bone reconstructions were based on WBCT scans that did not allow for visualization or segmentation of cartilage. Therefore, all joint parameter results (i.e. coverage, congruency, and joint space) should be interpreted accordingly, i.e., based on subchondral bone morphology alone. Second, the current study cannot describe sex-related morphometric variations of the talocrural joint. More participants are likely required before differences can be observed between sexes. Continued recruitment of healthy individuals is intended to build this normative dataset which can be used for future clinical comparisons to patients with hindfoot disease, deformity, and osteoarthritis. Third, the current SSM approach presented herein is a hybrid multi-articulation modeling approach. The SSM mean correspondence particles from individual bone SSM solutions are combined with the WBCT posture at time of image acquisition for each individual participant to report joint parameter results. With this modeling approach, it is not possible to draw SSM based conclusions regarding multi-domain modes of variation demonstrating the alignment of the bones with respect to each other. For our cohort of healthy individuals, participants were clinically screened to include individuals with a neutral hindfoot alignment, hence excluding anyone with deformities or mal alignment. For future studies including patients with concomitant deformities, a multi-domain SSM approach would be necessary to answer clinical questions regarding talocrural alignment. Ongoing computational development recently applied multi-domain SSM approaches to the pelvis and femur^40^. We intend to apply these new methods to patient populations that present with concomitant deformities in the future.

To conclude, we found that the ankle joint complex is highly congruent in healthy individuals with only minimal inter-individual morphometric differences of the articular regions. Such information is crucial to improve the current understanding of ankle or hindfoot pathologies and for further development of operative treatment strategies.

## Methods

### Data source and study population

Twenty-seven asymptomatic individuals were included in this study (age: 50.0 ± 7.3 years; height: 169.4 ± 6.4 cm; BMI: 25.3 ± 3.8 kg/m^2^; 7 males). Informed consent was obtained from all individual participants of this study. The study was carried out in accordance with the Declaration of Helsinki. Imaging data was previously collected at Kantonsspital Baselland, Switzerland with Institutional Review Board (IRB) approval (Ethics Committee Northwest/ Central Switzerland, BASEC 2016-01343). Data was collaboratively shared via a material transfer agreement (#5885) with the University of Utah under IRB approval (#65620). Inclusion criteria were individuals between 40 and 70 years of age without a history of ankle injury, surgery, or deformity. Exclusion criteria included individuals that presented with a planovalgus or cavovarus deformity based on clinical and radiographic assessment, screened by a board-certified orthopaedic surgeon. No individuals were excluded following screening by an orthopaedic surgeon.

### Computed tomography acquisition and surface generation

**Figure 7:**
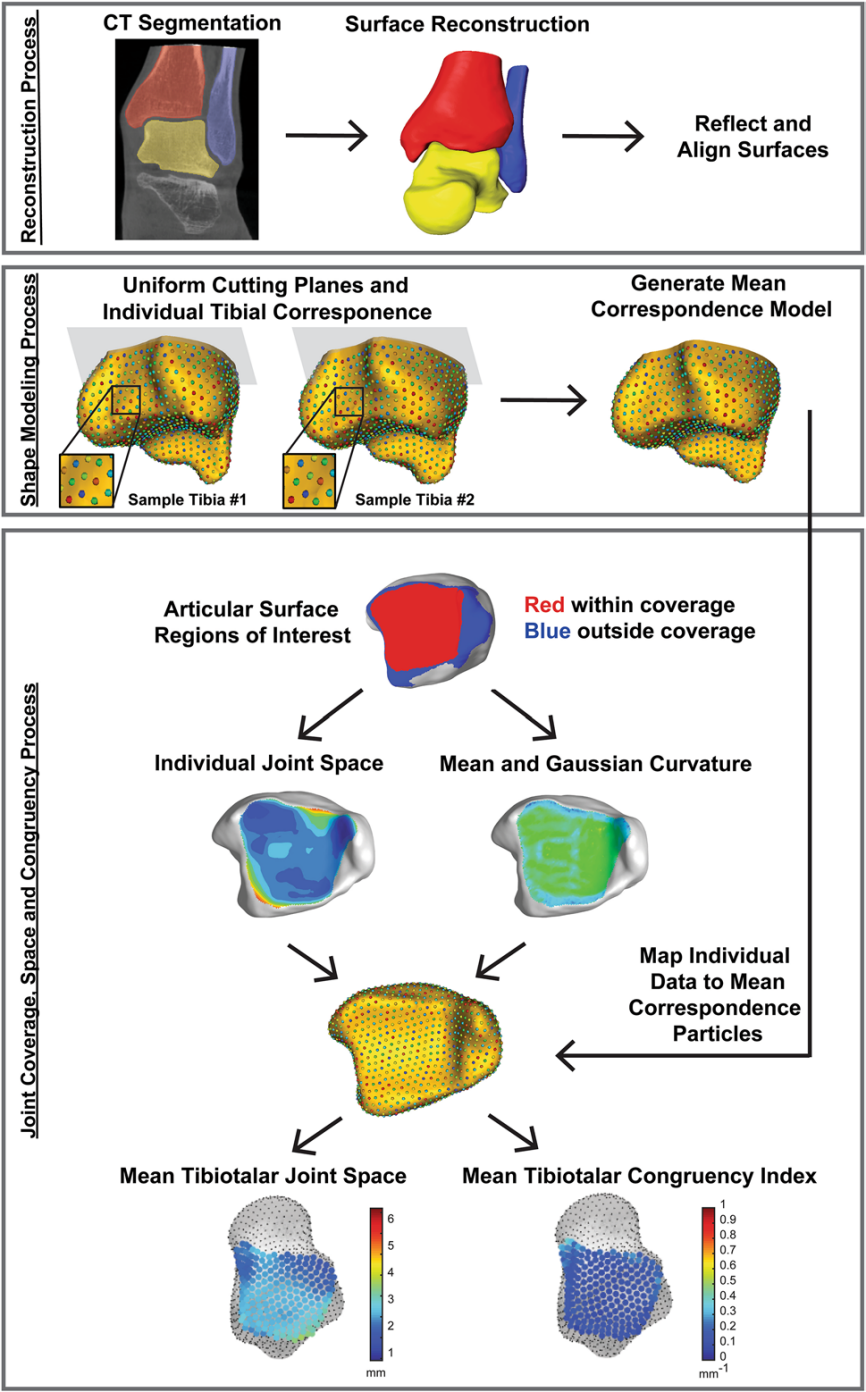
Flow chart of image and model processing. Subject-specific tibia, fibula and talus bone models were segmented and reconstructed from weightbearing computed tomography scans. Surfaces were reflected (all right limbs) and aligned. Cutting planes for the tibia (shown in grey) and fibula were uniformly applied and used to limit correspondence particle locations on the proximal axial plane. Correspondence particles are visualized as the multi-color points on the golden tibia models. Correspondence particles are shown in a detailed zoomed in view within the fibular notch on two patient specific tibiae (Sample Tibia #1 and #2). The zoomed in view of correspondence particles demonstrates the unique location of each particle that is consistently and mathematically placed on each bone specimen. For example, the grouping of green, blue, and red particles shown will maintain this ordered location across all bones and in the resulting mean correspondence model. Mean correspondence models were used in conjunction with joint level analyses of coverage, joint space distance and congruency for the tibiotalar (shown), tibiofibular and talofibular articular regions. Resulting joint parameters were averaged and visualized at correspondence particle locations within the articular regions.

Each participant underwent a weightbearing CT scan (WBCT) (Planmed Verity, Planmed Oy, Helsinki, Finland) with 0.4 mm slice thickness at 1 mm slice intervals. Participants stood in a single limb balanced position for imaging with the axis between their calcaneus and second metatarsal aligned parallel to the scanner’s anteroposterior axis. The field of view included the foot and ankle, including approximately 2 cm of the distal tibia. Alignment in the scanner was consistently performed with the individual’s calcaneus aligned in reference to the second metatarsal which was centered and parallel to the scanner’s anteroposterior axis. One limb was imaged per participant at random to avoid duplication of similar morphology represented in the dataset which would compound statistical testing of nested data. CT scans were segmented to create 3D surface models of the tibia, fibula, and talus (Amira, v6.0.1, Visage Imaging, San Diego, CA, USA) (Fig. 7). Surfaces of the tibia, fibula and talus were smoothed and decimated to reduce manual segmentation artifact and maintain consistent surface faces across the bone models.

### Correspondence-based statistical shape modeling

Pre-processing steps for SSM model development started with reflecting all surface meshes (.ply file format) representing left bones (tibia, fibula, talus) to appear as right bones. Then all bones were aligned using an iterative closest point algorithm, with the talus used as the reference bone to maintain a weightbearing alignment of the tibia and fibula with respect to the talus^41^. ShapeWorks^14^ was used to quantify anatomical variation of the bones of the talocrural joint (ShapeWorks, University of Utah; shapeworks.sci.utah.edu). The ShapeWorks workflow accepts the aligned binary image segmentations in the form of surface meshes as input, however, ShapeWorksRun performs the shape analysis and establishes correspondence on volumetric datasets in the form of distance transforms (.nrrd file format) using face indices (i.e., voxel-based representation of each 3D surface). Therefore, 3D surface meshes (.ply) were converted to distance transforms (.nrrd) during a preprocessing step called ShapeWorksGroom. Next, with the aligned distance transforms, a single cutting plane was identified for the tibia and fibula because the purpose of this study was to analyze anatomical differences in the articular regions associated with the talocrural joint. The single cutting plane for the tibia and fibula was identified perpendicular to the tibial shaft, proximal to the fibular notch at a mathematically standardized plane which corresponded to the most proximal uniform plane containing all aligned specimens (Fig. 7). Initially a correspondence model of 512 particles (i.e. landmarks) per bone was used to optimize the template plane used for all tibia and fibula pairs. Cutting planes were visually inspected for consistency across the dataset. The final model used 1,024 correspondence particles per bone (tibia, fibula, talus) that were hierarchically placed using a computational optimization splitting strategy below the cutting plane for all shapes using ShapeWorks^16,42,43^. The talus was a complete bone model and did not contain any distal cutting planes. Procrustes analysis was used to remove scaling (i.e. size) from the shape modeling analysis^44,45^.

Mean shapes and modes of variation for the tibia, fibula and talus were generated using ShapeWorks^14^. The correspondence particles locations were analyzed to define mean shapes, which were used to quantify differences in 3D anatomy. Principal component analysis (PCA) was used to reduce the locations of all correspondence particles (i.e., high-dimensional data) to a smaller set of linearly uncorrelated components, termed ‘modes’. Significant PCA modes were identified using parallel analysis in the R statistical software (https://www.R-project.org)^46^. For each significant mode for the tibia, fibula and talus, the mean surface model was exported and the mean surface was warped about ± 2 standard deviations (SD). To visualize anatomic differences within a mode of variation, surface distance color maps were calculated between the mean and warped ± 2 SD surface reconstructions in CloudCompare (v2.11.alpha, www.cloudcompare.org).

### Talocrural joint coverage, space and congruency

#### Coverage analysis

Analysis of articular coverage within the talocrural joint was separated into three regions of interest containing combinations of two mated surfaces: the tibiotalar (tibia and talus), tibiofibular (tibia and fibula), and talofibular (talus and fibula) regions. The coverage analysis was performed with the bones aligned in the original CT coordinate system which was defined at time of the image capture as a neutral weightbearing position. For each participant’s original bone reconstructions, the surface elements within three regions of interest for articulating surfaces of the talocrural joint (tibiotalar, tibiofibular and talofibular regions) were isolated using values of second principal curvature calculated in the PostView software (v2.1.0, FEBio, Salt Lake City, UT). The isolated areas of interest were then used to perform the coverage analysis. Coverage was defined as the surface area on each of the two mated articular surfaces that contained an intersection of normal vectors (Fig. 7). A tool in PostView identified these regions and calculated the surface area in each paired set of articular regions. The paired articular surface areas in the tibiotalar, tibiofibular and talofibular regions within the coverage area were statistically compared using a paired t-test followed by a post hoc power analysis (G*Power^47^) with a Cohen’s D^48^ calculation of observed effect size (small: d = 0.2, medium: d = 0.5, and large: d = 0.8).

#### Joint space distance analysis

Joint space distance calculations were performed on the identified articular surface coverage regions to calculate a Euclidian distance between the two articular surfaces at every node on the surface mesh (Fig. 7). This distance measure was conducted using the “Distance Map” tool in PostView which outputs a data field calculated for each node on one mated surface to the closest point distance to the opposing surface. It is important to note, this is a bone to bone distance and does not account for the presence of cartilage due to the limitations of imaging cartilage on a WBCT. The joint space distance at all patient specific surface mesh nodes was exported as a text file with surface node numerical identifiers and distance values at each node. Across the studied population, consistent locations were desired to conduct a population-based statistical analysis across the articular surface. This was desired to provide a joint space mapping of distance instead of reporting only a single joint space distance average within each articular region. To achieve this, each participant’s bone models were aligned using iterative closest point registration with the SSM mean talar or tibial shape to determine common correspondence particle locations in the articular regions. Identified correspondence particles used for the analysis included 228 locations on the tibiotalar articulation, 125 locations on the tibiofibular articulation, and 91 locations on the talofibular articulation. Joint space distance at each correspondence particle across the population were calculated and viewed using MATLAB. An average joint space distance and ± 1 standard deviation were plotted and reported visually at every correspondence particle. Lastly, joint space distances for the entire articular regions (tibiotalar, tibiofibular and talofibular) were averaged (± standard deviations) and a global minimum and maximum value across the entire population were reported.

#### Congruence index analysis

To mathematically present congruency (i.e. how well two surfaces conform), the congruence index (mm^-1^) was calculated based on the methods of Ateshian et al^49^. To begin congruency calculations, the same identified articular surface coverage regions (tibiotalar, tibiofibular, and talofibular) were used to calculate mean and Gaussian curvatures for all participants using PostView. The curvature at all patient specific surface mesh nodes was exported as a text file with surface node numerical identifiers and curvature values at each node. The same common correspondence particles that were used in the joint space distance calculations were used for the congruency analysis. At each correspondence particle, the calculation began with four values: mean curvature on surface one (*H*^*s1*^), mean curvature on surface two (*H*^*s2*^), Gaussian curvature on surface one (*G*^*s1*^), and Gaussian curvature on surface two (*G*^*s2*^).

Principal curvatures (*K*) for each surface (s1 or s2) were calculated as a minimum and maximum at each correspondence particle with Eqs. (1) through (4):

1 $${K}_{min}^{s1} = {H}^{s1} - \sqrt{{({H}^{s1})}^{2}- {G}^{s1}}$$

2 $${K}_{max}^{s1}= {H}^{s1}+ \sqrt{{({H}^{s1})}^{2}- {G}^{s1}}$$

3 $${K}_{min}^{s2}= {H}^{s2}- \sqrt{{({H}^{s2})}^{2}- {G}^{s2}}$$

4 $${K}_{max}^{s2}= {H}^{s2}+ \sqrt{{({H}^{s2})}^{2}- {G}^{s2}}$$

Curvature differences (*D*) were then calculated using Eqs. (5) and (6):

5 $${D}^{s1}=( {K}_{min}^{s1}- {K}_{max}^{s1} )$$

6 $${D}^{s2}=( {K}_{min}^{s2}- {K}_{max}^{s2} )$$

Relative Principal Curvatures ($${K}_{min}^{e} and {K}_{max}^{e})$$ were then calculated with respect to the two articular surfaces at each correspondence particle with Eqs. (7) and (8):

7 $${K}_{min}^{e}= {H}^{s1}+{H}^{s2}-\frac{1}{2} \Delta$$

8 $${K}_{max}^{e}= {H}^{s1}+{H}^{s2}+\frac{1}{2} \Delta$$

where ∆ is defined as the square root of Eq. (9); we applied the small-angle approximation^50^, which states that for small angles measured in radians: $cos\alpha \approx 1$. Due to the nature of our perpendicular selection of locations for paired articular surfaces at the correspondence particles, we can use this approximation to assume that α ≈ 0 (the angle between two points on the articular surfaces) and therefore $cos2\alpha \approx 1:$

9 $$\Delta^{2} = (D^{s1} )^{2} + (D^{s2} )^{2} + 2(D^{s1} *D^{s2} *cos2\alpha )$$

Therefore, perfect congruency at a single point of contact would result when ${K}_{min}^{e}= {K}_{max}^{e}=0$. Congruence Index calculations were performed as a root mean square (RMS) at each correspondence particle’s point of contact and defined in Eq. (10):

10 $${K}_{rms}^{e}= \sqrt{\frac{{({K}_{min}^{e})}^{2}+ {({K}_{max}^{e})}^{2}}{2}}$$

All values of Congruence Index reported herein are the result of Eq. (10) where ${K}_{rms}^{e}$ only reduces to a value of zero for perfectly congruent surfaces at a point. The Congruence Index at each correspondence particle across the population were calculated and viewed using MATLAB. An average Congruence Index and ± 1 standard deviation were plotted and reported visually at every correspondence particle. Lastly, Congruence Indices for the entire articular regions (tibiotalar, tibiofibular and talofibular) were averaged (± standard deviations) and a global minimum and maximum value across the entire population were reported (Fig. 7).

## Data Availability

The data generated during this analysis is available at Zenodo and can be used under the Creative Commons Zero v1.0 Universal license. Included is the dataset used to create statistical shape models utilizing ShapeWorks (https://github.com/SCIInstitute/ShapeWorks). Final segmented three-dimensional surface meshes (.ply) of weightbearing computed tomography (CT) images are included for the tibia, fibula and talus. A sample weightbearing CT scan (.dicom) is provided to demonstrate image resolution, field of view and voxel size. Additionally, the MATLAB code repository for calculating talocrural joint space and congruency is included with required files for duplicating the analysis. https://doi.org/10.5281/zenodo.4274217.
